# Adiponectin levels and its relation with insulin secretion and insulin sensitivity in a group of sub-Saharan African women with polycystic ovary syndrome

**DOI:** 10.1186/s13104-021-05878-0

**Published:** 2022-01-28

**Authors:** Audrey Synthia Momo, Vicky Jocélyne Ama Moor, Aurel T. Tankeu, Falmata Amazia, Guy Sadeu Wafeu, Magellan Guewo-Fokeng, Esther Astrid Mbono Samba, Jan réné Nkeck, Yannick Djieka, Christelle Chemaga Nkonpawa, Floriane Djapa Tofeun, Serge Guifo, Julius Dohbit Sama, Siméon Pierre Choukem

**Affiliations:** 1 Department of Biochemistry and Physiological Sciences, Faculty of Medicine and Biomedical Sciences, University of Yaoundé I, Yaoundé, Cameroon; 2 Laboratory of Biochemistry, University Teaching Hospital, Yaoundé, Cameroon; 3 Department of Biomedical Sciences, Faculty of Biology and Medicine, University of Lausanne, Lausanne, Switzerland; 4 Center for Research on Filariasis and Other Tropical Diseases, Yaoundé, Cameroon; 5 Department of Internal Medicine and Specialties, Faculty of Medicine and Biomedical Sciences, University of Yaoundé I, Yaoundé, Cameroon; 6 Health and Human Development Research Network, Douala, Cameroon; 7 Department of Obstetrics and Gynecology, Faculty of Medicine and Biomedical Sciences, University of Yaoundé 1, Yaoundé, Cameroon; 8 Department of Internal Medicine and Specialties, Faculty of Medicine and Pharmaceutical Sciences, University of Dschang, Dschang, Cameroon; 9 Department of Internal Medicine, Douala General Hospital, Douala, Cameroon

**Keywords:** Adiponectin, Polycystic ovary syndrome, Insulin sensitivity

## Abstract

**Objectives:**

Low levels of adiponectin have been reported in Polycystic Ovary Syndrome (PCOS). In sub-Saharan Africa, little data are available on the topic**.** We aimed to investigate the levels of adiponectin and its relation with insulin secretion and insulin sensitivity in women with PCOS in Yaoundé, Cameroon. A comparative cross-sectional study was conducted in 32 women presenting PCOS and 32 controls matched for age and Body Mass Index. For each participant, adiponectin levels were measured. We estimated insulin sensitivity using Homeostasis model index (HOMA-IR) and insulin secretion with C-peptide levels.

**Results:**

Women with PCOS had higher insulin secretion levels than controls (C-peptide: 4.98 ± 3.83 *vs* 3.25 ± 1.62 mUI/l; *p* = 0.02). Also, the HOMA-IR index was higher compared to that of women without PCOS (1.15 ± 0.90 *vs* 0.77 ± 0.38; *p* = 0.03) suggesting greater insulin resistance. The median [25th–75th percentile] values of adiponectin concentrations were similar between the two groups (22.68 [21.72–23.41] μg/ml *vs* 22.03 [21.40–22.93] μg/ml; *p* = 0.1). There was no association between insulin sensitivity and adiponectin levels in the PCOS group. PCOS is not associated with changes in adiponectin in a population of sub-Saharan African women. Further studies are needed to shed more light on this condition.

**Supplementary Information:**

The online version contains supplementary material available at 10.1186/s13104-021-05878-0.

## Introduction

Polycystic ovary syndrome (PCOS) is a complex and heterogenous condition characterized by hyperandrogenism, ovulatory dysfunction and polycystic ovarian morphology (PCOM) [1, 2]. It affects women of reproductive age worldwide with an estimated prevalence of 5–20% [1]. Metabolic abnormalities, mainly insulin resistance (IR) and compensatory hyperinsulinemia, are evident in a majority of affected individuals, especially among those women who also show hyperandrogenism [1]. It has been shown that 50–70% of women with PCOS are insulin resistant [3], half of them are overweight or obese with 5–7-folds increased risk of Type 2 diabetes (T2D) [4]. The mechanisms of IR in this condition remain poorly elucidated. Initially, IR was considered a consequence of obesity and overweight usually found in PCOS. However, recent evidence suggests that insulin resistance in PCOS might be independent of obesity [5]. In search of mechanisms and associated factors of IR, adipokines were proposed as a potential actor in the development of IR in PCOS [6]. Adiponectin is one of the most abundant adipokine, plays an important role in energy metabolism and contributes to the pathogenesis of the metabolic syndrome [6, 7]. It also exhibits anti-inflammatory, anti-atherogenic and insulin-sensitizing effects [8]. Reduced levels of adiponectin have been associated with insulin resistance indicating a potential role in the development of metabolic disorders [9]. Given that, it has been suggested that adiponectin levels might be altered in PCOS participating to IR in this condition. However, this relationship between adiponectin levels and IR in PCOS remains debatable. Studies reports opposing results with some studies finding lower adiponectin levels in PCOS independent of BMI [10–12], and others reporting similar adiponectin levels in BMI-matched PCOS and controls [13, 14]. But while these data are accumulating in different populations, data on this issue are lacking on sub-Saharan African populations. Therefore, studies are needed to provide data on this topic on a population where metabolic disorders are highly prevalent [15]. In this light, we aimed to study the levels of adiponectin in cameroonian women living with PCOS.

## Main text

### Materials and methods

#### Design/participants

This comparative cross-sectional study was conducted from March to June 2020 at the Yaoundé Gynaeco-Obstetric and Paediatric Hospital and at the laboratory of the Yaoundé Central Hospital. We included women of reproductive age (15–44 years) living with PCOS and controls matched for age and BMI (ratio 1:1). PCOS was defined according to the 2003 Rotterdam diagnosis criteria of PCOS; we therefore included in the PCOS group, women with at least 2 of the following criteria: (1) oligo-or anovulation; (2) clinical and/or biological hyperandrogenism; (3) polycystic morphology of ovary in ultrasound examination (at least 12 follicles with a diameter of 2 to 9 mm and/or volume ≥ 10 ml per ovary) [16]. Control subjects were healthy women recruited in the community, with no menstrual cycle disorders and no signs of clinical hyperandrogenism. We did not include women with any of the following conditions: pregnancy or breastfeeding, known diabetes mellitus, known chronic diseases or other hyperandrogenemia conditions (late-onset congenital adrenal hyperplasia, Cushing syndrome, androgen secreting tumors, and thyroid dysfunction). In addition, oral contraceptives or drugs that could affect hormonal and metabolic profiles were discontinued at least 3 months before inclusion in the study.

#### Data collection

For each subject, we measured weight with an automatic scale (CAMRY®, Hong Kong, China) and Height with a stadiometer. Waist circumference (WC) and hip circumference (HC) were measured using a measuring tape. We calculated Waist-to-hip ratio (WHR) as $\frac{\mathrm{WC}}{\mathrm{HC}}$ and body mass index (BMI) using the Quetelet’s formula as BMI = weight/height^2^ (kg/m^2^). We grouped participants into 3 categories according to BMI: normal BMI (18.5–24.9 kg/m^2^), overweight (25.0–29.9 kg/m^2^) or obese (≥ 30 kg/m^2^) [17]. We measured resting blood pressure using standardized procedures with a validated automated blood pressure measuring device, Omron HEM-757(Omron Corporation, Tokyo, Japan).

#### Blood samples and laboratory investigations

After twelve hours of overnight fast, 10 ml of venous blood were collected in the morning on the antecubital vein in dry tube and sodium fluoride tube. Fasting plasma glucose levels were determined the same day by an enzymatic colorimetric method using BIOLABO® kits (France). Serum was then aliquoted and stored at − 20 °C for further biochemical analysis (C-peptide and adiponectin). C-peptide and adiponectin were measured by an indirect sandwich Enzyme Linked Immuno-Sorbent Assay (ELISA) method (ELABSCIENCE® kits, USA).

#### Determination of insulin resistance

We used the Homeostasis Model Assessment of Insulin Resistance (HOMA-IR). This was determined by the formula: Fasting blood glucose (mmol/l) × Fasting insulin (µUI/ml)/22.5) where insulin was replaced by the C-peptide level as previously published in our context [18, 19].

#### Statistical analysis

Data were analyzed using SPSS version 23.0 software (IBM Corporation, Chicago, Illinois, USA). For quantitative variables, results are presented as mean ± Standard Deviation (SD) for normally distributed data or median (25th–75th percentiles) when normality was not verified. For categorical variables, results are presented as counts (percentages). We used the Chi-square test to study association between categorical variables. The Student’s *T*-test and non-parametric test (Mann–Whitney *U*-test) were used for group’s comparison of quantitative variables. Spearman’s correlation test was used to assess the linear association between skewed quantitative data and logistic regression to assess independent variables associated with PCOS. The receiver operating characteristic (ROC) curve analysis was used to evaluate the performance of adiponectin and Waist-to-hip ratio (WTH) in predicting PCOS. The significance threshold was set at p < 0.05.

### Results

#### Characteristics of the study population

Overall, we enrolled 32 women with PCOS with a mean age of 26.7 ± 4.7 (20–38) years. Of these women, 31.3% had normal BMI, 31.3% were overweight and 37.4% were obese. The general characteristics of participants are summarized in (Table 1).

**Table 1:** Clinical characteristics of participants

Variables	PCOS n = 32	Controls n = 32	*p*
Age (years)	26.7 ± 4.7 (20–38)	27.1 ± 4.9 (18–38)	0.73
Body mass index (Kg/m2)	28.9 ± 1.4 (15.6–51.4)	28.3 ± 1.2 (19.5–46.9)	0.72
Normal BMI	10 (31.3)	10 (31.3)	1
Overweight	10 (31.3)	10 (31.3)	1
Obese	12 (37.4)	12 (37.4)	1
Waist circumference (cm)	92.3 ± 3.5 (64–126)	87.02 ± 2.9 (66–131)	0.25
Waist-to-hip ratio	0.93 ± 0.02 (0.70–1.18)	0.82 ± 0.01 (0.65–0.97)	** < 0.001**
Systolic blood pressure (mm Hg)	118.3 ± 2.08 (102–160)	117.7 ± 2.03 (90–139)	0.84
Diastolic blood pressure (mm Hg)	77.2 ± 1.8 (60–120)	79.1 ± 1.5 (58–107)	0.41

Values are count (percentages), mean ± SD (range)

Test in bold represent significant values

#### Insulin resistance and adiponectin levels

Women with PCOS had higher C-peptide levels compared to the controls (4.98 ± 3.83 *vs.* 3.25 ± 1.62 mUI/l; *p* = 0.02), whereas their fasting plasma glucose levels were similar (5.27 ± 1.05 *vs.* 5.35 ± 0.94 mmol/l; *p* = 0.75). As a result, their HOMA-IR index was higher compared to that of women without PCOS (1.15 ± 0.90 *vs.* 0.76 ± 0.38; *p* = 0.03). Serum levels of adiponectin were similar between the two groups (Table 2).

**Table 2:** Biochemical characteristics of participants

Variables	PCOS n = 32	Controls n = 32	*p*
Adiponectin (μg/ml)	22.68 [21.72–23.41]	22.03 [21.40–22.93]	0.1
Fasting plasma glucose (mmol/l)	5.27 ± 1.05	5.35 ± 0.94	0.75
C-peptide (mUI/l)	4.98 ± 3.83	3.25 ± 1.62	**0.02**
HOMA-IR index	1.15 ± 0.90	0.77 ± 0.38	**0.03**

Values are median [25th–75th percentile] or mean ± SD

Test in bold represent significant value

In women with PCOS, we didn’t find any correlation between adiponectin levels and the HOMA-IR index (*p* = 0.23) on one hand, nor between adiponectin levels and C-peptide, fasting plasma glucose, or BMI on the other hand (Table 3). After logistic regression analysis, we found that Waist-to-hip ratio was independent factor associated with PCOS (Additional file 1: Table S1). Waist-to-hip ratio presented with the best power in predicting PCOS (AUC = 0.76) followed by adiponectin (AUC = 0.62) (Additional file 1: Fig. S1).

**Table 3:** Correlation between adiponectin levels and HOMA-IR index, C-peptide, fasting plasma glucose, Body mass index

Variables	Adiponectin levels (μg/ml)			
	PCOS		Controls	
	r	*p*	r	*p*
HOMA-IR index	− 0.19	0.23	0.03	0.89
C-peptide (mUI/l)	− 0.15	0.40	− 0.04	0.82
Fasting plasma glucose (mmol/l)	− 0.23	0.20	0.13	0.48
BMI	0.11	0.82	0.32	0.08

### Discussion

PCOS is frequently associated with IR leading to compensatory hyperinsulinemia [1]. In the present study, women with PCOS had a high HOMA-IR index compared to matched controls suggesting greater insulin resistance. These results are consistent with those of Amer et *al*. in Egypt where cases were more insulin resistant than controls using the same index [10]. In addition, Doh et *al*. in their report in fourteen Cameroonian women with PCOS, also found a low level of insulin sensitivity using the euglycemic hyperinsulinemic clamp, the reference method for estimating insulin sensitivity [20]. The C-peptide levels obtained, reflecting insulin secretion, were significantly higher in the PCOS group than controls suggesting hyperinsulinemia in this population as expected. In women with PCOS, basal insulin secretion rates are increased, although insulin secretory responses to a glucose load are generally inadequate resulting in a lower glucose disposition index than age and BMI-matched controls [21]. Despite the presence of hyperinsulinemia, women with PCOS are thought to have pancreatic β­cell dysfunction and also demonstrate decreased hepatic clearance of insulin [22]. Insulin potentiates steroidogenic response to gonadotrophins both in vivo and in vitro; hence, during hyperinsulinemia there will be elevated androgen levels. This increase androgen activity is associated with IR [23]. It has been hypothesized that PCOS results from a vicious circle of androgen excess favoring abdominal adipose tissue deposition and visceral adiposity by inducing IR and compensatory hyperinsulinism, which further facilitates androgen secretion by the ovaries and adrenal glands in women with PCOS [24].

Adiponectin values obtained in PCOS women fall within the known normal range of 5-30 µg/mL and were similar to the controls group [9]. Some studies report similar results in BMI-matched PCOS and controls [13, 14] whiles other report low levels of adiponectin independent of BMI [10–12]. As in our study, the authors did not find a relation between adiponectin levels and insulin resistance [11]. They attributed this changes to fat distribution and variable amount of subcutaneous and high visceral fat [11, 25]. In our study, we found that WTH was independent factor associated with PCOS and have the best discriminatory power in predicting PCOS with an AUC of 0.76. One study showed that it can be used as indirect predictors of visceral obesity in women with PCOS [26]. Therefore, this abdominal obesity could cause additional disturbances in metabolic and hormonal parameters in PCOS [27]. The comparable results between PCOS and controls in our study suggest a relatively increase level of adiponectin in our population. Considering that this is the first study in this population, we speculate that this relative increase in adiponectin may reflect a resistance to its action [28]. This can either be a low sensitivity to this hormone, or a post-receptor resistance leading to a compensatory mechanism increasing hormonal levels.

We did not found a relation between adiponectin levels and insulin sensitivity in women with PCOS. Similar results were reported in Poland population [29]. However, studies in the Caucasian population report an association between adiponectin and insulin sensitivity [12, 30]. These discrepancies between our findings and reports in other populations further stress the need of studies on the question in sub-Saharan populations.

### Conclusion

Sub-Saharan African women with PCOS do not present lower adiponectin levels despite higher insulin resistance and high insulin secretion levels. Further studies are needed to shed more light on this condition.

## Limitations

Firstly, we used C-peptide measurement to access insulin level base on their equimolar secretion in blood. Therefore, it can be indirectly measured to access insulin secretion [31]. Secondly, HOMA-IR index was used to determine insulin sensitivity instead of the gold standard method (the euglycaemic hyperinsulinic clamp) used in a small number of subjects [32]. Although, studies recommend the use of HOMA-IR index when it is impossible or difficult to perform [33]. In addition, the HOMA-IR index is more suitable for population and epidemiological studies [18].

## Supplementary Information

**Additional file 1: Table S1.** Logistic regression including key independent variables and factors associated with PCOS. **Figure S1.** Receiver operating characteristic (ROC) curve was based on binary logistic regression and classification analysis for PCOS and control group. AUC: Area under the curve.

## Data Availability

Data will be available from the corresponding author upon request.

## Abbreviations

BMI: Body mass index
HC: Hip circumference
HOMA-IR: Homeostasis Model Assessment of Insulin Resistance
PCOS: Polycystic ovary syndrome
IR: Insulin resistance
SD: Standard deviation
T2D: Type 2 diabetes
WC: Waist circumference
WHR: Waist to Hip Ratio
AUC: Area under the curve
ROC: Receiver operating characteristic
