# Supplementary material for: Adiponectin levels and its relation with insulin secretion and insulin sensitivity in a group of sub-Saharan African women with polycystic ovary syndrome
Source: BMC Res Notes. 2022 Jan 28;15:24. doi: 10.1186/s13104-021-05878-0 (PMC8796504; doi:10.1186/s13104-021-05878-0)
Supplement: Supplementary file 1 — Additional file 1: Table S1. Logistic regression including key independent variables and factors associated with PCOS. Figure S1. Receiver operating characteristic (ROC) curve was based on binary logistic regression and classification analysis for PCOS and control group. AUC: Area under the curve. [file 13104_2021_5878_MOESM1_ESM.docx]

**Additional file 1:**

Table S1: Logistic regression including key independent variables and factors associated with PCOS

| **Variables** | **Beta coefficient (B)** | **Exp (B)** | **95% CI for Exp (B)** | | ***p*** |
| --- | --- | --- | --- | --- | --- |
| Adiponectin (ug/mL) | 0.288 | 1.334 | 0.700 | 2.543 | 0.382 |
| Homa-IR | -0.416 | 0.660 | 0.015 | 28.662 | 0.829 |
| C-Peptide (pg/ml) | 0.016 | 1.016 | 0.974 | 1.061 | 0.462 |
| BMI (kg/m2) | -0.102 | 0.903 | 0.815 | 1.002 | 0.054 |
| Waist-to-hip ratio | 12.641 | 309061.832 | 164.281 | 581438374 | **0.001** |


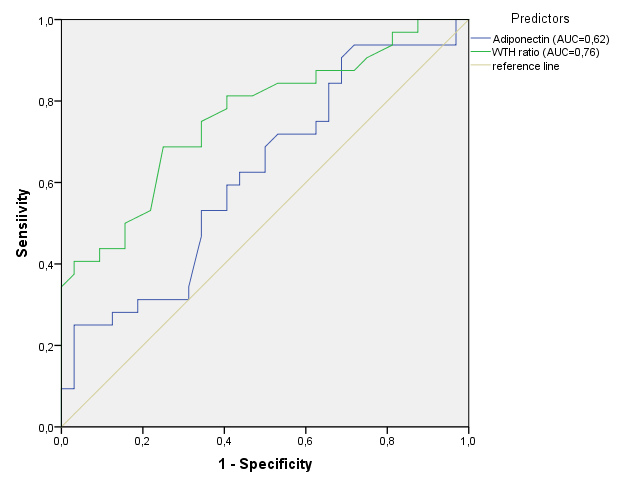


**Fig. S1:** Receiver operating characteristic (ROC) curve was based on binary logistic regression and classification analysis for PCOS and control group. AUC: Area under the curve.
